# Proinflammatory Cytokines Are Elevated in Serum of Patients with Multiple System Atrophy

**DOI:** 10.1371/journal.pone.0062354

**Published:** 2013-04-23

**Authors:** Eli Kaufman, Sara Hall, Yulia Surova, Håkan Widner, Oskar Hansson, Daniel Lindqvist

**Affiliations:** 1 Department of Clinical Sciences, Section for Psychiatry, Lund University, Lund, Sweden; 2 Department of Clinical Sciences, Lund University, Lund, Sweden; 3 Department of Neurology, Skåne University Hospital, Lund, Sweden; 4 Clinical Memory Research Unit, Department of Clinical Sciences, Lund University, Malmö, Sweden; 5 Psychiatry Skåne, Lund, Sweden; Philadelphia VA Medical Center, United States of America

## Abstract

**Background:**

Despite several lines of evidence from preclinical and post-mortem studies suggesting that inflammation is involved in Multiple System Atrophy (MSA), no previous studies have measured peripheral indices of inflammation in MSA patients.

**Methods:**

We measured C-reactive protein, interleukin (IL)-6, soluble IL-2 receptor and tumor necrosis factor (TNF)-α in blood samples from MSA patients (n = 14) and healthy controls (n = 40).

**Results:**

IL-6 and TNF-α were significantly elevated in MSA patients compared to healthy controls. After controlling for the potentially confounding effects of age, gender, and somatic co-morbidities, a diagnosis of MSA was still significantly associated with high levels of TNF-α. Higher TNF-α levels were associated with less severe motor symptoms and earlier disease stage.

**Conclusions:**

Our findings are in line with the hypothesis that inflammation might be involved at an early stage of MSA pathophysiology.

## Introduction

Multiple System Atrophy (MSA) is an adult-onset, neurodegenerative disease with poor prognosis and unknown cause. MSA is clinically characterized as a variable combination of parkinsonism, cerebellar ataxia, autonomic dysfunction and/or corticospinal tract impairment [1].

The neuropathological hallmark is the presence of α-synuclein containing glial cytoplasmic inclusions in oligodendrocytes in the striatonigral and/or the olivopontocerebellar pathways [2]. Other consistent findings are neuronal destruction, astrogliosis, myelin loss and axonal degeneration [2].

Based on animal and neuropathological studies, neuroinflammation has been implicated in the pathophysiology of MSA. Activated microglia is a common finding in post-mortem brain tissue of MSA patients [3], and a PET study with a ligand for activated microglia marker PK 11195 demonstrated in vivo activation [4]. Moreover, results from a transgenic mouse model of MSA suggest an association between early and progressive microglial activation in substantia nigra and dopaminergic neuronal loss. On the basis of these and other findings, Ahmed et al. recently proposed a working hypothesis in which aberrant α-synuclein leads to degenerative changes, which in turn activate microglia to release proinflammatory and cytotoxic substances further exacerbating the degeneration – thus creating a vicious cycle [2]. However, only a few studies have investigated such proinflammatory substances in the context of MSA. In a post-mortem study, Schwartz et al. showed increased nuclear translocation of RelA, a subunit in the NF-κB family of proteins, in activated microglia of MSA brains – thus suggesting transcription of proinflammatory factors [5]. A clinical trial with the microglia stabilizing agent minocycline has been conducted, albeit with negative results [6]. We have not been able to retrieve any clinical investigations of peripheral inflammatory biomarkers in MSA patients. The main aim of this study was therefore to explore such biomarkers in serum of MSA patients and in healthy controls. Since high levels of pro-inflammatory cytokines have been associated with more severe symptoms of depression and fatigue in patients with Parkinson’s disease [7], we also wanted to investigate associations between such symptoms and cytokines in MSA patients. To this end, we measured C-reactive protein (CRP), TNF-α, IL-6 and soluble interleukin-2 receptor (sIL-2R) in the blood of MSA patients and healthy controls, all assessed for symptoms of depression, anxiety, fatigue and sleep difficulties.

## Methods

### Ethics Statement

The Ethics Committee of Lund University approved this study. All study participants gave written consent for participation in the study, which was performed in accordance with the provisions of the Helsinki Declaration.

### Study Participants

Between 2008 and 2012, 14 MSA patients and 40 healthy controls gave written and informed consent to participate in the study. The study received approval from the Regional Ethical Review Board in Lund. All patients were recruited from different neurological clinics in southern Sweden, while most healthy controls were spouses of patients or otherwise part of their extended family. The study procedures took place at Skåne University Hospital in Lund. A complete medical history was taken and routine blood work conducted, followed by a clinical evaluation to verify the diagnosis as either probable (n = 11) or possible (n = 3) MSA according to the consensus statement [1]. Ten patients were diagnosed with predominant parkinsonian symptoms (MSA-P) and 4 with predominantly cerebellar ataxia (MSA-C). Subjects with dementia, acute or chronic inflammatory disease or ongoing treatment with NSAIDs or corticosteroids were excluded from the study. During the visit, a licensed and experienced medical doctor evaluated the subjects using the Unified Parkinson’s Disease Rating Scale (UPDRS)-3 [8], the Hoehn & Yahr staging scale [9], and the Schwab & England activities of daily living (ADL) scale [10]. The UPDRS-3 includes a clinical examination of motor symptoms of PD, the range is 0–56 points, higher score representing more severe disability. The Hoehn & Yahr scale divides patients into stages on the basis of clinical disability. Finally, the Schwab & England scale evaluates degree of independence where a score of 0 is bedridden, with no swallowing, bowel or bladder function and 100 represents complete independence. Demographic characteristics of patients and controls are given in Table 1.

**Table 1:** Demographic characteristics and cytokine levels of patients and controls.

	Controls (n = 40)	Patients (n = 14)	p-value
Sex	females = 26 (65%), males = 14	females = 8 (57%), males = 6	.60^a^
Age *(years, mean ± SD)*	64.8±9.0	64.8±8.6	.99^c^
Illness duration *(years, median, IQL)*		6, 5–10	
Hoehn and Yahr *(mean ± SD)*		4.0±089	
Schwab & England *(median, IQL)*	100, 100–100	45, 30–80	.00^b^
UPDRS motor score *(median, IQL)*	0, 0–2,	42, 25–59	.00^b^
CRP *(median, IQL)*	1.3,.6–2.4	1.5,.7–4.5	.59^b^
IL-6 *(median, IQL)*	2.8, 2.8–2.8	3.0, 2.8–7.0	.005^b^
sIL-2R *(mean ± SD)*	427.0±131.6 *(n = 39)*	463.8±150.9	.39^c^
TNF-α *(median, IQL)*	9.5, 7.0–11.8	11.0, 9.0–15.3	.012^b^
HAD-anxiety *(median, IQL)*	1.0,.0–6.0 *(n = 38)*	8.5, 5.3–12.3	.001^b^
HAD-depression *(median, IQL)*	.0,.0–2.0 *(n = 39)*	5.5, 1.8–9.8	.001^b^
FACIT-fatigue *(median, IQL)*	51.0, 49.0–52.0 *(n = 36)*	28.0, 15.5–39.0 *(n = 11)*	.001^b^
SCOPA-sleep night *(median, IQL)*	3.0, 1.0–5.0 *(n = 39)*	5.5, 3.5–9.3	.03^b^

a Pearson’s chi-square.

b Mann-Whitney U-test ^c^Student’s t-test.

IQL = inter quartile Range; S = standard deviation.

CRP = C-reactive protein; IL-6 = Interleukin-6; sIL-2R = soluble interleukin-2 receptor; TNF-α = tumor necrosis factor (TNF)-α; HAD = Hospital Anxiety and Depression Scale; FACIT = Functional Assessment of Chronic Illness Therapy; SCOPA = Scales for Outcomes in PD; UPDRS = Unified Parkinson’s Disease Rating Scale.

The study participants were asked to fill in the Functional Assessment of Chronic Illness Therapy-Fatigue (FACIT-F) [11], Hospital Anxiety and Depression Scale (HAD) [12] (including subscales for symptoms of depression as well as anxiety) and Scales for Outcomes in PD-Sleep (SCOPA-S) [13]. A cut-off value of 8 in the HAD subscales has been suggested to have optimal specificity and sensitivity in finding cases with an anxiety or depression disorder [14]. HAD and SCOPA scores were available from all patients, while FACIT-F scores were only available from 11 patients. For SCOPA-S, only the score of nighttime sleeping difficulties was used in the statistical analyses.

### Blood Sampling and Analysis

The blood samples were obtained by drawing 5 ml serum for immediate analysis at the department of clinical immunology at Skåne University Hospital in Lund. Chemiluminescent assays (Immulite 1000 Siemens) were used. Serum was incubated with monoclonal antibodies coated on polystyrene beads for 30 minutes; thereafter a polyclonal anti-antibody labeled with alkaline phosphatase was added before another 30 minutes incubation. Unbound conjugate was washed away and chemiluminescent substrate added. After ten minutes, light emission was measured and concentrations calculated using samples with known concentrations as references. The detection limits were 2.8 ng/L for IL-6, 1.7 ng/L for TNF-α, 5 kU/L for sIL-2R and 0.6 mg/L for CRP. In the entire sample, 38 (70.4%) of the subjects had IL-6 levels below detection limit, and were assigned the value 2.8. In the same manner, 12 (22.2%) of the subjects had CRP levels below 0.6 mg/L, and were subsequently assigned the value 0.6. None of the other biomarkers were outside of detection range.

### Statistics

The Statistical Package for the Social Sciences (SPSS) for Mac was used for statistical calculations. CRP, IL-6, and TNF-α, were all non-normally distributed, hence the Mann-Whitney U-test was used for group-wise comparisons and Spearman’s rho for correlative analyses. Levels of sIL-2R were normally distributed; hence Students T-test was used for group-wise comparisons. Pearson’s chi-squared test was used to compare proportions. When comparing cytokines and clinical variables between patients with MSA-P and MSA-C non-parametric tests were used due to the small number of cases in each group. In order to be able to answer the question whether the relationships between MSA and high levels of IL-6 and TNF-α were confounded by age, gender or somatic illness, we conducted two separate linear regression analyses with lnIL-6 and lnTNF-α entered as dependent variables in each respective analysis. Age, gender, MSA diagnosis (dichotomous variable; MSA diagnosis yes/no), and somatic illness were all entered as independent variables in each regression model. Somatic illness was calculated as a continuous variable based on the occurrence of the most common somatic co-morbidities, all known to be able to influence cytokine levels. Those were: Cardiovascular disease (MSA patients N = 4 = 29%; Controls N = 13 = 33%), asthma/allergies (MSA patients N = 1 = 7%; Controls N = 5 = 13%), osteoarthritis (MSA patients N = 1 = 7%; Controls N = 4 = 10%), and diabetes mellitus (MSA patients N = 2 = 14%; Controls N = 1 = 3%). The continuous variable was calculated as a composite score of all somatic co-morbidities for each individual in order to capture total burden of somatic illness. P-values below 0.05 were considered significant.

## Results

Compared with controls, patients with MSA had significantly higher levels of IL-6 (Mann-Whitney U = 167.0, p = .005) and TNF-α (Mann-Whitney U = 153.0, p = .012), but not sIL-2R (t(51) = –0.86, p = 0.39, ns) or CRP (Mann-Whitney U = 253.0, p = .59, ns).

MSA patients suffered from more severe non-motor symptoms than controls, as measured with the four self-assessment scales. There were significant differences in the scores on FACIT-F (Mann-Whitney U = 18.5, p<.001), HAD anxiety (Mann-Whitney U = 102.5, p = .001), HAD depression (Mann-Whitney U = 91.5, p<.001) and SCOPA-S (Mann-Whitney U = 168.0, p<.05). Using a cut-off value of 8 for the HAD subscales, there were significant differences in the number of subjects having a high degree of anxiety; 57% of the MSA sufferers compared with 16% of the controls (Pearson’s c^2^ = 9.6, p<.01). Similarly, 36% of the MSA sufferers had a high degree of depressive symptoms compared with 5% of the controls (Pearson’s c^2^ = 8.7, p<.01).

There were no significant correlations between any of the biomarkers and the non-motor symptoms in the MSA group (Spearman’s rho, all p-values >.06). TNF-α was significantly and negatively correlated with Hoehn & Yahr staging scale scores (Spearman’s Rho = –0.59, p = .003), significantly and positively correlated with Schwab & England ADL scale scores (Spearman’s Rho = 0.61, p = .002), and significantly and negatively correlated with UPDRS-3 scores (Spearman’s Rho = −0.70, p = .0005). None of the other cytokines correlated significantly with any of the symptom scales (Spearman’s rho, all p-values>.06).

There were no significant differences in any of the cytokines or clinical characteristics between patients with MSA-P and patients with MSA-C (Table 2).

**Table 2:** Demographic characteristics and cytokine levels in patients with MSA with predominant parkinsonism (MSA-P) and MSA with cerebellar ataxia (MSA-C).

	MSA-P (n = 10)	MSA-C (n = 4)	p-value
Sex	females = 7 (70%), males = 3	females = 1 (25%), males = 3	.12^a^
Age *(years, median, IQL)*	65, 59–71	68, 51–72	.89^b^
Illness duration *(years, median, IQL)*	7, 5–10	5, 4–6	.20^b^
Hoehn and Yahr *(median, IQL)*	4, 3–4	5, 4–5	.12^b^
Schwab & England *(median, IQL)*	45, 38–80	45, 23–75	.52^b^
UPDRS motor score *(median, IQL)*	42, 24–60	43, 27–58	.96^b^
CRP *(median, IQL)*	1.3,.6–2.9	2.9,.9–4.7	.48^b^
IL-6 *(median, IQL)*	3.0, 2.8–7.0	2.9, 2.8–6.0	.71^b^
sIL-2R *(median, IQL)*	469, 409–632	405, 289–493	.26^b^
TNF-α *(median, IQL)*	11.0, 9.8–15.5	12.0, 8.3–15.8	.62^b^
HAD-anxiety *(median, IQL)*	7.0, 2.5–11.5	11.0, 7.0–15.0	.20^b^
HAD-depression *(median, IQL)*	4.5, 1.0–7.5	10.0, 5.8–15.8	.08^b^
FACIT-fatigue *(median, IQL)*	29.5, 14–46 *(n = 8)*	22.0, 8.0–28 *(n = 3)*	.18^b^
SCOPA-sleep night *(median, IQL)*	4.0, 3.3–8.5	8.0, 3.3–10.5	.39^b^

a Pearson’s chi-square.

b Mann-Whitney U-test.

IQL = inter quartile range; SD = standard deviation.

CRP = C-reactive protein; IL-6 = Interleukin-6; sIL-2R = soluble interleukin-2 receptor; TNF-α = tumor necrosis factor (TNF)-α; HAD = Hospital Anxiety and Depression Scale; FACIT = Functional Assessment of Chronic Illness Therapy; SCOPA = Scales for Outcomes in PD; UPDRS = Unified Parkinson’s Disease Rating Scale.

In the regression model with lnTNF-α entered as the dependent variable, and MSA diagnosis, age, gender, and somatic illness entered as independent variables, the overall model was significant (adjusted R square = 0.26, p<0.001). MSA diagnosis was significantly associated with lnTNF-α (beta = 0.41, p<0.001) whereas age, gender, and somatic illness were not (data not shown). When lnIL-6 was entered as a dependent variable, the overall model was not significant (adjusted R square = 0.004, p<0.39), and MSA diagnosis did not reach significance in the regression model (beta = 0.25, p = 0.08). However, one of the controls was an extreme outlier with regards to IL-6 levels, thus IL-6 levels was not normally-distributed even after log transformation. After excluding the extreme outlier, the overall model was significant (adjusted R square = 0.12, p<0.035), and MSA diagnosis was significant also in the regression model with lnIL-6 as dependent variable (beta = 0.39, p = 0.004). Age, gender, and somatic illness were not, however, significantly associated with lnIL6 in any of these regression models (data not shown).

## Discussion

Our results indicate that MSA patients have significantly higher serum levels of IL-6 and TNF-α, two key inflammatory markers, compared to healthy controls. The association remained significant for TNF-α even after controlling for the potentially confounding effects of age, gender, and somatic co-morbidities. When patients were split into groups based on their main symptomatology, cerebellar versus parkinsonian, there were no significant differences in cytokine levels between the two groups. High cytokine levels thus seem to be associated with the MSA diagnosis, rather than a specific subtype of the disease.

Our findings are well in line with the reports of microglial activation in vivo and in post-mortem examinations of MSA patients [3], [4]. Activated microglia are known to express both IL-6 and TNF-α among other cytokines and chemokines [5]. Interestingly, a genetic study suggested that TNF-α is involved in the pathophysiology of MSA [15]. We recently reported that patients with Parkinsońs disease (PD) have significantly higher median serum levels of IL-6 than healthy controls [7]. Conversely, no significant difference in TNF-α levels was found in that study. This suggests that the increased IL-6 levels observed in the present study may be non-specific for MSA, whereas high TNF-α levels may in fact distinguish MSA patients from PD patients. Post-hoc analyses comparing cytokine levels between the present study sample and the one previously described [7] revealed a trend for a significant difference in TNF-α levels between MSA and PD patients (Mann-Whitney U = 421.0, p = .07), whereas the difference in IL-6 was non significant (Mann-Whitney U = 488.0, p = .21).

Chronic inflammation in the central nervous system can lead to neurodegeneration [16]. Results from some animal- and in vitro studies can be translated into the hypothesis that neuroinflammation is an early feature and causally linked to MSA development, leading to neuronal loss. This was explored in a clinical trial with minocycline [6]. The negative result of that study could be related to treatment initiated too late, or with an imperfect mechanism of drug action. We found significant correlations between TNF-α and motor symptom severity as measured by Hoehn & Yahr staging scale, Schwab & England ADL scale and UPDRS-3. Interestingly, our data indicates that the patients with more severe motor symptomatology in fact display lower levels of TNF-α; findings that are in line with the notion that neuroinflammation may be an early event in MSA pathophysiology [2], [17]. To the best of our knowledge, this is the first study to investigate correlations between cytokines and severity of MSA. Similar studies in PD have yielded different results; Scalzo et al. found no significant correlations between serum IL-6 and PD symptom severity (UPDRS-3, Hoehn & Yahr, and Schwab & England) [18], while Menza et al. found a positive correlation between TNF-α and Schwab & England ADL scale scores, but not with UPDRS [19]. In line with the results of the present study, Müller et al. found a significant inverse correlation between IL-6 in cerebrospinal fluid and UPDRS I-III in PD patients [20].

This seemingly contra-intuitive relationship between higher cytokine levels and less severe symptoms and earlier disease stage could in fact strengthen the inflammatory hypothesis of MSA pathogenesis. Müller et al. argues that elevated cytokine levels in the early stage of neurodegeneration could reflect a compensatory physiologic response to neuronal damage, with potential neuroprotective purposes [20]. These associations could also suggest that neuroinflammation is specifically related to pathophysiological processes in MSA, as opposed to a more general sign of aging, health decay, or burden of illness.

MSA patients suffer from more severe symptoms of depression, anxiety, fatigue and sleeping difficulties than healthy controls do, most likely as a consequence of the degenerative processes as well as reactive depression. The actual prevalence of these symptoms in our material is high but in line with the survey of quality of life issues in MSA where a similar fining of 46% was reported [21].

In conclusion, our results indicate that MSA patients have increased serum levels of some pro-inflammatory cytokines compared with controls. Higher cytokine levels seem to be associated with less severe symptomatology, which may suggest that neuroinflammation is an early event in MSA pathology. Our findings add to the small but quite solid body of evidence suggesting a role for neuroinflammation in the pathophysiology of MSA. However, further prospective studies need to confirm our findings.
